# A framework for transformational leadership to enhance teacher’s work performance

**DOI:** 10.3389/fpsyg.2024.1331597

**Published:** 2024-07-23

**Authors:** Xia Yu, Guanwei Jang

**Affiliations:** ^1^Management School, Guangdong University of Science and Technology, Dongguan, Guangdong, China; ^2^Business School, Shaoguan University, Shaoguan, Guangdong, China

**Keywords:** transformational leadership, organizational vision, inspiring communication, intellectual stimulation, supportive leadership, personal recognition, moral modeling, teachers’ work performance

## Abstract

**Introduction:**

In the new era of higher education focusing on “double first-class” development, private universities in China face significant opportunities and challenges. The success of these institutions is closely linked to effective leadership, particularly transformational leadership, which is known to inspire and motivate employees toward extraordinary achievements. This study explores the existence and impact of transformational leadership on teachers’ work performance in Chinese private universities. It aims to fill a research gap regarding the role of transformational leadership in a resource-constrained environment and provide practical insights for enhancing teachers’ work performance, thereby contributing to the rapid development of private universities and the realization of “double first-class” goals.

**Methods:**

This study employed an input-process-output approach, combining qualitative data from face-to-face interviews with 12 leaders at Guangdong University of Science and Technology and quantitative data from an electronic survey of 392 university teachers. Data were analyzed using SPSS26.0, AMOS24.0, and NVIVO14, including exploratory and confirmatory factor analyses, common method bias assessment, and correlation analysis. Structural equation modeling with AMOS24.0 was used to explore the relationship between transformational leadership and teachers’ work performance, evaluating the impact of each transformational leadership dimension.

**Results:**

The study findings demonstrated that transformational leadership styles are being employed in Chinese private universities, albeit not extensively, with moderate effects on teachers’ work performance. The research identified organizational vision, intellectual stimulation, and personal recognition as significant drivers of enhanced performance among faculty members. Nevertheless, the study did not confirm the impact of supportive leadership, inspiring communication, and moral modeling within the context of Chinese private universities.

**Discussion:**

The results highlight areas for potential development in leadership practices, including improving teachers’ competencies, motivating teachers, providing good work opportunities and environments, and building good teacher teams.

## 1 Introduction

In the new period, with the deployment of the higher education focusing on “double first-class” (The implication is that first-class universities and first-class disciplines construct), private universities have ushered in new development opportunities. However, the development of HEIs is inextricably linked to leaders (Kalsoom et al., 2018). They must face the complex environment of change and guide employees to achieve the organization’s goals through various ways (Shao et al., 2022). The role of transformational leaders for organizations and individuals is well known (Lawrason et al., 2023). The theory suggests that transformational leaders fulfill the personal needs of their followers, motivate and inspire their followers to achieve extraordinary things, and develop their leadership skills (Bass and Riggio, 2006). Leaders with this leadership style can create a positive organizational culture (Jaskyte, 2004) that promotes creativity (Koh et al., 2019), innovation, and growth (Gumusluoglu and Ilsev, 2009). They can communicate a clear organizational vision and motivate followers to work toward achieving that goal (Givens, 2008). They can also provide followers with personalized support and guidance to help them develop their skills and reach their full potential (Alimo-Metcalfe and Alban-Metcalfe, 2001). Begum et al. (2018) suggested that transformational leadership styles can be used to study prevalent leadership styles, performance, and effectiveness in organizations such as the higher education sector. Liu (2018) examined the development of transformational leadership theories in China using 233 Chinese transformational leadership literature and pointed out the need to make further efforts to study the indigenous transformational leadership practices that are suitable for China’s unique cultural context of education. Moreover, existing studies in other areas on the impact of transformational leadership on employee performance provide conflicting empirical evidence (Buil et al., 2019). For example, the findings of Kammerhoff et al. (2019) showed that transformational leadership has a significant effect on employee performance. However, the findings of Eliyana and Ma’arif (2019) reached just the opposite conclusion. More interestingly, a recent study that questioned and examined the effectiveness of transformational leadership behaviors for all followers found that not all followers uniformly embrace transformational leadership behaviors (Seitz and Owens, 2021). Therefore, the researchers would like to know whether transformational leadership style exists in the management of private universities in China? What is the current status of teachers’ work performance in private universities? Can transformational leadership have a positive effect on teachers’ work performance in Chinese private universities in a resource-disadvantaged environment? The exploration of these questions will help us better understand the characteristics of transformational leadership in Chinese private universities, which not only fills a gap in the research on transformational leadership and teachers’ work performance in private universities, but also provides referable practical operations for private universities to improve teachers’ work performance, promote the rapid development of private universities, and realize the construction goal of “double first-class.”

## 2 Theoretical basis and research hypotheses

### 2.1 Theoretical basis

This section described a synthesis of research on transformational leadership, teachers’ work performance, IPO approach, and AMO theory to provide a theoretical basis for the conceptual framework and research hypotheses.

#### 2.1.1 Transformational leadership (TFL)

TFL is a broadly inclusive concept. Burns (1978) argued that leaders are seen as transformative when they support and encourage followers to improve their morality, motivation, beliefs, cognitive skills, and connection with corporate goals (Bass et al., 1987; Metwally et al., 2014). Bass (1985) believed that TFL is the process by which a leader changes the values and beliefs of his subordinates and guides them to go beyond their self-interests to pursue higher goals. And TFL was divided into charismatic-inspirational leadership, intellectual stimulation and individualized consideration. Later, Bass (1996) further distinguished charismatic-inspirational leadership into two dimensions: charisma and inspiration. In this way, the four-dimensional structure of TFL was obtained: charisma or idealized influence, inspirational motivation, intellectual stimulation and individualized consideration.

As the concept of TFL has evolved, an increasing number of scholars have offered different perspectives. Rafferty and Griffin (2004) revisited the theoretical model developed by Bass (1985) and argued that it is vision, not leadership charisma, that is the real reason for attracting employees to action; that inspirational communication is a unique dimension of facilitating vision success; supportive leadership is included in the construct of personalized care, which expresses the leader’s respect for followers and concern for their feelings and needs; and personal recognition is used to express the contingent rewards associated with TFL. To identify the five sub-dimensions of change were demonstrated to differentiate the effectiveness of leadership among each other as well as on outcomes. Based on Bass et al.’s (1997) findings, domestic scholars Li-Chaoping (2005) further explored the specific dimensions of TFL in the Chinese context, adding the dimension of moral modeling while retaining the original dimensions of Charisma, articulate vision, and individualized consideration. Finally, the four dimensions of TFL are formed in line with the characteristics of Chinese culture. In summary, this paper constructs a six-dimensional concept of TFL: organizational vision, inspirational communication, intellectual stimulation, supportive leadership, personal recognition, and moral modeling. And redefined as follows:

TFL is a cluster of six distinct but related behaviors. Leaders convince employees through their noble ethics, quality, and behavioral examples. who use inspirational communications to communicate a shared organizational vision and efforts to instill a sense of pride in their followers. And demonstrate an appreciation and understanding of followers’ preferences, values, needs, and concerns to highlight employee traits, accomplishments, and strengths as supportive leadership to followers. Moreover, these leaders are adept at using praise and rewards to recognize employees’ efforts. Building on this good leadership-membership relationship, leaders encourage employees to question traditional practices (e. g., traditional teaching methods) and come up with creative, novel problem-solving to drive organizational change. In the process of this interaction, gain more mature and excellent leaders and followers.

#### 2.1.2 Teachers’ work performance (TWP)

Work performance is one of the most important indicators of employee outcomes. It includes both the results of direct tasks and some behaviors that promote high-quality task completion and contribute to the sustainability of the organization. In Borman and Motowidlo (1997) proposed the theory of individual differences in work performance and divided it into task performance and contextual performance. Later, adaptive performance was also categorized as a dimension of work performance (Griffin et al., 2007). In addition, the learning performance dimension of work performance was also proposed by London and Sessa (2006) and (Han et al., 2007). Considering the existing theories of work performance and the duties of teachers in Chinese universities, this paper categorizes TWP into learning performance (LP), innovative performance (IP), work dedication (WD), helping and collaborating (HOTC), and teacher-student interaction (TSI). And defines it as a collection of school-related outcomes and behaviors presented by teachers within a certain time or space. It is mainly reflected in two aspects: teaching and scientific research, including learning subject knowledge, teaching methods, and teaching theories; actively declaring projects, caring for students, and communicating with students; leading and guiding students to participate in various competitions, etc.

#### 2.1.3 Input-process-output (IPO)

Input-output analysis is a type of project that first originated in economics as a sort of bringing a balance between demand and both the volume and quality of supply. This type of analysis was then implemented in several other fields including education. The term IPO is defined as “inputs that lead to processes that in turn lead to outcomes” (Ilgen et al., 2005, p. 519), which enables categorizing variables, relationships and patterns, and research gaps (De Massis et al., 2013; Strike et al., 2018).

#### 2.1.4 Ability-motivation-opportunity (AMO) theory

The AMO framework is based on organizational psychology, which consists of three basic concepts: the individual competencies and skills needed to achieve superior performance (Ability); the motivation to achieve the desired performance (Motivation), and the environmental and situational constraints provided by the organization related to performance (Opportunity). Some scholars have tried to explain performance by linking it to TFL (Li et al., 2020). The main idea of these studies is (A) to address skill gaps in achieving the vision by communicating a shared vision for the organization. These solutions include providing employees with training, leadership modeling, IS, or relying on other organization-specific skills as well as some transferable skills (e.g., digital technology), etc. Then, transformational leaders use PR and IC as a way to increase employee motivation (M). Finally, transformational leaders use their professional judgment, experiential coaching, etc. to support employees and recognize their individual needs to ensure that employees are given more opportunities (O) to participate in the organization. When leaders prepare their employees to achieve specific goals, employees can exceed expectations. The framework for the above exposition can be seen in Supplementary Figure 1.

#### 2.1.5 Conceptual framework

**FIGURE 1:**
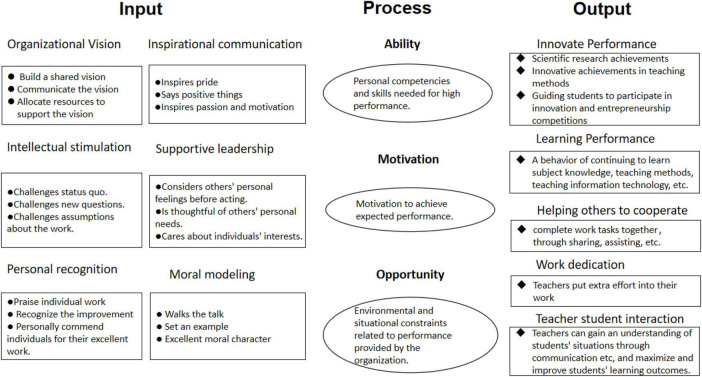
Conceptual framework.

Based on the above literature analysis, combined with the IPO approach, we can try to use AMO as a link between TFL and TWP to construct the conceptual framework of this paper as shown in Figure 1.

### 2.2 Research hypotheses

#### 2.2.1 Transformational leadership in higher education institutions (HEIs)

The literature review found that the characteristics and role of TFL in HEIs have received attention from some scholars. Zhang et al. (2009) took an ordinary institution of higher learning in Shandong Province as an example. The investigation of TFL behavior found that transformational leaders not only have the characteristics of exemplary morality, vision incentives, individualized consideration, and leadership charm but also have the characteristics of decision-making, coordination-ability, solidarity, and people-oriented. These characteristics show a trend of diversification and intersection. The descriptive statistical analysis of the questionnaires showed that the teachers in the school had better working conditions and emotional responses. Huang et al. (2021) studied how department chairs affect teachers’ organizational commitment and found that psychological empowerment mediated the relationship between TFL and organizational commitment as well as contingent reward leadership also positively impacted teachers’ organizational commitment. Al-Husseini and Elbeltagi (2018) compared the impact of TFL on innovation in private and public HEIs in Iraq and found that the relationship was stronger in public HEIs. Al-Husseini et al. (2021) continued their study in public HEIs in Iraq and found that TFL could not have a direct impact on faculty innovation but rather supported innovation through knowledge sharing. These studies show that TFL exists and plays a role in higher education management, hence the hypothesis of this study:

H1:TFL has been displayed in private universities in China.

#### 2.2.2 Transformational leadership and teachers’ work performance

Previous studies have shown that high-level TFL supervisors have a positive impact on employee job performance (Judge and Piccolo, 2004; Piccolo and Colquitt, 2006; Bono et al., 2007). Lai et al. (2020) also found that TFL behaviors have a positive impact on employee performance. Top et al. (2020) used Bass’s four-dimensional structure of TFL to test the TFL effect of employees and managers in shopping malls in Erbil and Sulaymaniyah in Kurdistan, and found that motivation and personalized care have a significant effect on employee performance, while idealization and intellectual stimulation have a weaker effect on employee performance. An empirical study by Andriani et al. (2018) showed that TFL has a positive and significant impact on teacher performance. In China, Li-Chaoping (2005) pointed out that among the four dimensions of TFL style, leadership charisma and individualized care have a prominent impact on employees’ job performance, and among them have the greatest impact on work dedication; Song (2010) survey found that individualized care, morality, vision motivation, and leadership charisma have an impact on job performance, but not all correlations are very significant (Wenyi, 2016). Through interviews with younger teachers in universities, it was found that the behavior of the TFL can improve the bad emotions of younger teachers caused by work pressure, promote the growth of younger teachers more effectively, and improve their work performance. Guan (2020) conducted a study on the work performance of administrators in universities, and the results showed that TFL promotes the work performance of administrators in universities. Employee identity shapes a good organizational climate, which in turn reinforces the positive impact of TFL on job performance. It can be seen that TFL can drive teachers’ internal factors thereby improving their performance. In summary, this study proposes the following hypothesis:

H2: TFL has a positive impact on TWP in private universities in China.

#### 2.2.3 Organizational vision (OV)

OV refers to an idealized picture of the future based on organizational values (Rafferty and Griffin, 2004). It is not essentially a goal, but an idea worth pursuing for a long time. It represents the idea, image and mental description of the organization’s future (O’Connell et al., 2011) and can have a positive impact on individual employees. Many researchers have noted that a vision communicated throughout an organization is a key factor in organizational success, and those entities without a vision “stumble in the dark” (Jantz, 2017). OV has a cohesive, motivational, orientational, and normative role, which stimulates the organization as a whole to engage in creative and collaborative learning to enhance organizational learning (Jisun, 2009). Organizational mission and vision affect individual performance (Dermol and Širca, 2018) and also have a positive impact on employees’ innovative performance (Slåtten et al., 2021; Shao et al., 2022). Research has shown that in the interaction between transformational leaders and employees, the organizational vision encourages subordinates to participate in organizational construction and do things outside of their roles that are beneficial to the organization, which improves performance. Therefore, this study proposes the following hypothesis:

H2a: OV of transformational leaders has a positive impact on TWP in private universities in China.

#### 2.2.4 Inspirational communication (IC)

IC is the expression of positive and encouraging messages about the organization, as well as statements that build motivation and confidence (Rafferty and Griffin, 2004). In the social psychology literature, researchers have shown that inspiration is associated with a range of positive outcomes, including improved wellbeing (Thrash et al., 2010; Salas-Vallina et al., 2020), promotion of multidisciplinary team innovation (Mitchell and Boyle, 2019), and innovation and entrepreneurship (Manuel, 2021), improving teachers’ teaching competence (Singh, 2019), and teachers’ normative commitment to work (Rukundo and Akurut, 2021). In addition, Salas-Vallina et al. (2020) stated that inspiration may be a key factor in improving followers’ skills and competencies. Tamsah et al. (2021) confirms the idea that inspirational leadership can improve the quality of employees’ work through soft skills and job innovation. Therefore, this study proposes the following hypothesis:

H2b: IC of transformational leaders has a positive impact on TWP in private universities in China.

#### 2.2.5 Intellectual stimulation (IS)

IS refers to leaders encouraging their followers to explore new ways of doing things and new learning opportunities. The findings of Çekmecelioğlu and Özbağ (2016) suggest that TFL behaviors are used to energize employees through IS and thus develop individual creativity. Koh et al. (2019) in a recent meta-analytic review stated that IS of leaders may have an indirect effect on followers’ creativity through followers’ creative identities, intrinsic motivation for creativity, psychological empowerment, creative self-efficacy, identification with the leader, and creative climate indirectly influencing followers’ creativity. From the AMO theoretical perspective, the intellectual stimulation of transformational leadership can solve employee competency gaps and thus provide the basis for good employee performance (Li et al., 2020). Therefore, this study proposes the following hypothesis:

H2c: IS of transformational leaders has a positive impact on TWP in private universities in China.

### 2.2.6 Supportive leadership (SL)

SL refers to a leader supporting each subordinate in striving for higher performance by providing emotions, information, trust, and tools, and helping him or her solve work-related problems (Rafferty and Griffin, 2004). In this way, leaders can help their subordinates avoid stress. It is the leader’s job to understand the needs of his or her employees and to address those needs appropriately. A workplace filled with supportive leaders leads to successful outcomes, which are beneficial to the wellbeing of both the employee and the organization. SL should be associated with creativity and innovation evidence (Lee et al., 2020). The study found a stronger relationship between SL and creativity in cultures with higher power distance. Analyzed from the AMO theoretical perspective, supportive leadership by transformational leaders can help employees achieve high performance by providing them with opportunities to achieve their goals (Li et al., 2020). Therefore, this study proposes the following hypothesis:

H2d: SL of transformational leaders has a positive impact on TWP in private universities in China.

#### 2.2.7 Personal recognition (PR)

PR refers to leaders recognizing followers’ performance through praise, rewards, etc. Followers identify with the leader’s behavior and actively strive to feed back the leader’s approval. This recognition can also be referred to as employee recognition in organizational management. Gilbert and Kelloway (2018) argued that recognition produces bidirectional positive outcomes between leaders and followers and demonstrated that recognition by transformational leaders has a strong relationship with employee wellbeing. Although there is not an abundance of literature that focuses directly on recognition (Gilbert and Kelloway, 2018), there are some conclusions that can be drawn from the existing literature that PR can be used as a tool for effective leadership because it motivates employees, builds positive relationships, and thus increases employee satisfaction, and performance (Conzelmann, 2020). Therefore, this study proposes the following hypothesis:

H2e: PR of transformational leaders has a positive impact on TWP in private universities in China.

#### 2.2.8 Moral modeling (MM)

MM refers to leaders who have a high ethical code of conduct, can lead by example, and are admired, respected, and trusted by their followers. Followers, on the other hand, identify with the actions of leaders and emulate them (Bass and Riggio, 2006). It emphasizes that leaders should have good moral character. Li-Chaoping (2005) pointed out that the most effective way to govern a country in China is to lead by example and virtue, to set an example for subordinates, and to influence them through subtle influence. Song (2010) found that the higher the degree of moral modeling, the higher the employee job satisfaction. In the correlation between job satisfaction variables and job performance variables, the higher the job satisfaction, the higher the situational performance. Therefore, this study proposes the following hypothesis:

H2f: MM of transformational leaders has a positive impact on TWP in private universities in China.

## 3 Methodology

**FIGURE 2:**
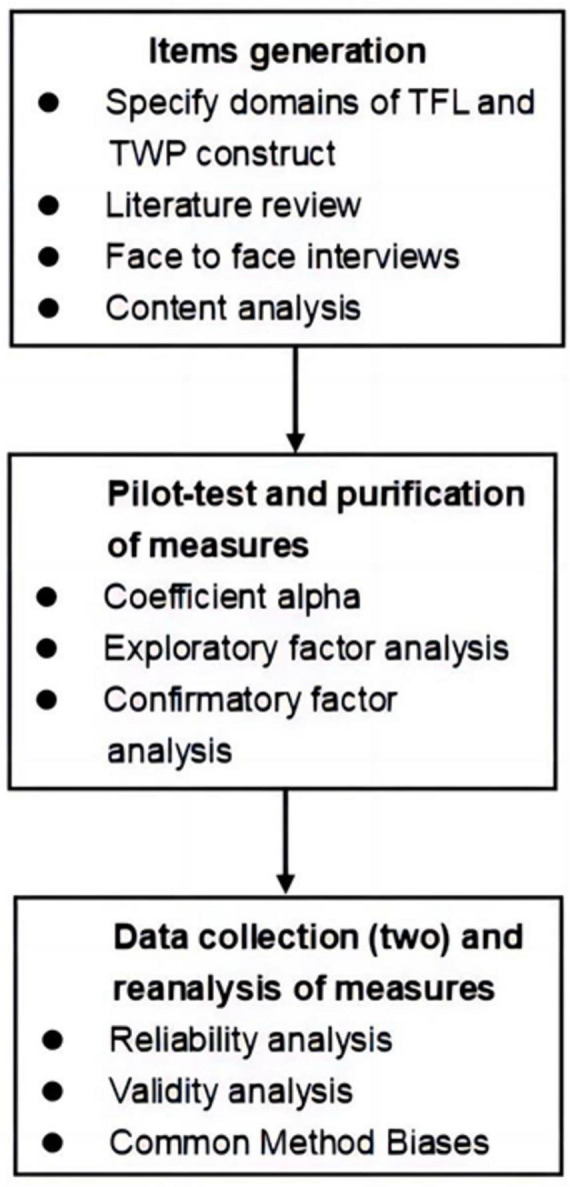
Flow chart of the scale development procedure.

In the study, the steps used for scale construction were primarily in accordance with the paradigm advocated by Churchill (1979) and Carlson et al. (2000). This paradigm is not only simple and clear but also widely used in academia. The development process for the TFL and TWP scale consisted of the following three steps: (a) initial item generation, (b) Phase 1 of data collection and purification of measures, and (c) Phase 2 of data collection and reanalysis of measures (Figure 2).

### 3.1 Items generation

#### 3.1.1 Specify domains of TFL and TWP construct

Churchill (1979) stated that during the early development of a scale, the scope of definitions should be specified. During this step, the study conducted a literature review and divided TFL into six dimensions on the basis of Rafferty and Griffin (2004): organizational vision, inspirational communication, intellectual stimulation, supportive leadership, personal recognition and moral modeling. TWP was categorized into five dimensions on the basis of Dai et al. (2017): innovation performance, learning performance, helping others to cooperate, work dedication and teacher student interaction. The study collected data from a wide range of literature and compiled a preliminary list of items that may be relevant to TFL and TWP. First, the characteristics of TFL and TWP were explored in the relevant literature, the dimensions of both were defined, and questionnaire items were collected from the literature related to TFL and job performance; second, in addition to adapting the dimensions of TFL from Rafferty and Griffin (2004), the study referred to the individual TFL items in Podsakoff et al. (1990), Bass (1996), House (1998), and Li-Chaoping (2005) to identify suitable items from existing scales. The questionnaire for TWP took into account the characteristics of the teacher profession in addition to the questionnaire of Dai et al. (2017). A total of 40 questionnaire items had been obtained at the end of this step.

#### 3.1.2 Face-to-face interviews

The literature review could not fully cover the content of TFL and TWP. To systematically understand the content or direction of TFL and TWP, the study invited 12 leaders at different levels working in private universities to conduct in-depth interviews.

All in-depth interviews followed a well-accepted protocol in order to standardize the interview results (Eisenhardt, 1989; Yin, 2009). Accordingly, an interview protocol was developed and was pretested with two academics specializing in organizational development research (Yin, 2009). Based on their feedback, the wording of some questions was slightly refined. In the next step, each interviewee received a file with a brief introduction, the purpose of this study, a guarantee of confidentiality, and a request for a 90-min interview. Each interviewee was asked six questions as follows:

1. In the process of organizational change, what leadership behaviors do you think will have some impact on subordinates?
2. Based on your work experience, what do you think is effective in motivating teachers?
3. As a leader, how would you treat your employees?
4. What criteria do you think organizations should use to evaluate the work performance of a university teacher? Why?
5. What do you think of organizational vision, inspirational communication, intellectual stimulation of employees, personal recognition of employees, support of employees, and the role of personal moral role models for teachers?
6. In the reform process, what do you think which aspects should be focus as a private universities’ leader?

The researcher had to make an appointment with the leader in advance because the leader was very busy. So, the interviews were conducted from early March 2023 until mid-June. A total of three department chairs, five deans, one HR manager, one director of academic affairs, one vice principal, and one principal were invited to participate in the interviews, which took an average of 46 min each.

#### 3.1.3 Content analysis

After the face-to-face interviews had been completed, the study converted the voice recordings into transcripts for subsequent data analysis. The study followed the recommendations of Kassarjian (1977), using content analysis and systematically analyze and organizing the interview data. NVIVO is a professional qualitative data analysis software that can help researchers to carry out in-depth thematic analysis and content analysis of unstructured data such as text, audio and video. First, all the audio data was imported into the system, and then the software used natural language processing technology to listen to the uploaded audio to generate accurate transcripts, ready for encoding and analysis. It saved valuable time for researchers. This study was mainly transcribed using NVIVO14 software, and obtained 15 analysis units through content analysis, mainly focused on all dimensions of TFL. 15 new questionnaire items were added to this study, and the total number of questionnaire items increased to 55 once this step was completed.

#### 3.1.4 Item content validity

After the initial items for the scale were developed, a panel of experts was recruited to assess the content validity of the scale and to evaluate its content and relevance. The panel consisted of one experienced leader and three academics specializing in management and organizational psychology. They did not participate in face-to-face interviews or content analyses. McCoach et al. (2013) noted that questionnaire items must be modified or deleted when items in a single dimension exhibit insufficient internal consistency. Therefore, the panel assessed the appropriateness of the questionnaire items in relation to the operational definitions and expressed their views on changing the wording. For example, for the TFL item, it was straightforward to change him/her to leader so that it would be easier for the respondents to understand. Other experts suggested that some of the question items were not very differentiated and that the wording needed to be changed. This panel of experts only gave suggestions for question item modifications and did not delete any items. The initial questionnaire is shown in Table 1.

**TABLE 1:** The initial questionnaire.

Dimension	Items	Relevant literature
OV of TFL	1. I know the development direction of the organization.*	House, 1998
	2. I know how far our unit will develop in five years.	
	3. Leader makes me feel that the organizational vision can increase my confidence in the organization.	Face to face interview
	4. I realize that my work is in line with the vision and goals of the company.	
	5. I know what we should do to achieve our vision and goals.	
IC of TFL	6. Leader will always say something that makes me feel proud as a member of the organization.*	Rafferty and Griffin, 2004
	7. Leader will always actively evaluate the work unit.	
	8. Leader will always encourage people to see the changing environment as a challenge full of opportunities.	
	9. The evaluation of the company from my leader makes me feel hopeful.*	Face to face interview
	10. Leader will always say something encouraging when new tasks come up.	
IS of TFL	11. Interacting with leader makes me feel challenged to think about old problems in new ways.*	Bass, 1996
	12. Interacting with leader makes me feel forced to rethink some things that have never been questioned before.	
	13. Interacting with leader makes me feel challenged to rethink some of my basic assumptions about my work.	
	14. Interacting with leader makes me feel challenged to seek differing perspectives when solving problems.	
	15. Interaction with leader makes me feel that I can question some of my supervisor’s work assumptions.*	Face to face interview
SL of TFL	16. Leader considers my personal feelings before acting.	House, 1998; Rafferty and Griffin, 2004
	17. Leader behaves in a manner which is thoughtful of my personal needs.	
	18. Leader sees that the interests of employees are given due consideration.	
	19. Leader can sincerely help me solve some difficulties in my work.*	Face to face interview
	20. Leader makes me feel like I’m being treated fairly.*	
PR of TFL	21. Leader will compliment me when I do my job better than average.	Podsakoff et al., 1990
	22. Leader will admit (affirm) the improvement of my work quality.	
	23. Leader praises me publicly when I do a good job.	
	24. Leader encourages employees to participate in important decisions.*	Face to face interview
	25. Leader always entrusted with important tasks to me.*	
MM of TFL	26. Tries his/her best at work regardless of personal interest.	Li-Chaoping, 2005
	27. Put one’s benefits after the group or organization’s benefits.*	
	28. Never take the achievements of other peoples as his/her own.	
	29. Never make things hard for his/her subordinates; never retaliate his/her subordinate by abusing his/her power.	
	30. Keeps learning for self-enhancement.	Face to face interview
IP of TWP	31. I often check the latest academic literature in the subject area.	Dai et al., 2017
	32. I actively apply for research topics or publish papers.	
	33. I pay attention to the development of the subject area closely.	
	34. I often take the initiative to undertake challenging teaching (new courses) and scientific research tasks.	
	35. I often take the initiative to lead students to participate in subject, industry technology or innovation competitions.*	
LP of TWP	36. I often eagerly impart new knowledge and new theories in this subject to students.	Dai et al., 2017
	37. I often update the teaching content according to the development of the subject.	
	38. I often learn to use new research methods or teaching methods.	
	39. I often adjust and use more effective teaching methods according to the situation of the students.	
	40. I often learn to use teaching information technology to improve teaching management.*	Face to face interview
HOTC of TWP	41. I often share teaching methods or skills with other teachers.	Dai et al., 2017
	42. I am often invited by other teachers to participate in the application of scientific research projects.*	
	43. I often provide good suggestions for the development of the school or the department.	
	44. I often promote or recommend my school on various occasions.	
	45. I often take the initiative to assist other teachers in completing various tasks.*	
WD of TWP	46. I often assume teaching or research tasks that are not my own. *	Dai et al., 2017
	47. I often voluntarily participate in work-related training or academic conferences.	
	48. I often take the initiative to solve problems at work without shirk.	
	49. Even during the holidays, I will carefully complete the review of students’ papers and homework.	Face to face interview
	50. Even after class, I will seriously answer the questions asked by the students.*	
TSI of TWP	51. I often communicate with students to understand their thinking and learning.	Dai et al., 2017
	52. I often take the initiative to communicate with students and answer questions.	
	53. I look for various ways to let students concentrate in the class.*	
	54. I take the initiative to help students with learning difficulties complete the courses.	
	55. I think of various ways to let students actively participate in various learning tasks.*	Face to face interview

*items deleted after EFA and CFA.

### 3.2 Pilot-test and purification of measures

#### 3.2.1 Exploratory factor analysis

To increase questionnaire reliability and validity, the researcher selected Guangdong University of Science and Technology (GDUST) for pilot administration. In the initial questionnaire of TFL and TWP, the 55 initial items obtained in the previous step were scaled using a 5-point Likert scale ranging from 1 (strongly disagree) to 5 (strongly agree) for subsequent surveying. They used personal chat tools like WeChat and QQ to distribute the questionnaire to colleagues, receiving 95 questionnaires in total. Four invalid questionnaires were removed due to factors such as response time and contradictory demographic information, leaving 91 valid questionnaires with an effective rate of 95.78%. The pilot testing allowed for any issues or errors to be identified and addressed before the survey was widely distributed.

To reduce the number of items and simplify the scale, the study conducted an exploratory factor analysis (EFA). The data were analyzed using dimensionality reduction analysis with reliability using SPSS 26.0 software. In this step, the criteria for deleting an item were (a) a factor loading of < 0.5 and (b) if the item exhibited a high factor loading on another factor (Hair et al., 2010). The EFA results revealed that the Kaiser–Meyer–Olkin value of TFL and TWP were 0.907 and 0.885; the approximate chi-square distribution were 2,475.612 and 1,803.972; and the degree of freedom (df) were 435 and 300, achieving the level of statistical significance (*p* < 0.01). Through this simplification process, the 55 initial items were reduced to 53 items with sufficient explanatory power.

#### 3.2.2 Confirmatory factor analysis

In this step, the study conducted a confirmatory factor analysis (CFA) to verify the validity and reliability of the pilot data. For the six factor models of TFL, the Cronbach’s alpha of the 20 items ranged between 0.879 and 0.925 (17 items TWP is ranged between 0.709 and 0.917), and the composite reliability (CR) of the latent variables ranged between 0.912 and 0.953 (TWP is ranged between 0.813 and 0.937). Both criteria exceed the threshold (0.70) suggested in Bagozzi and Yi (1988) and Hair (2009), indicating the excellent internal consistency of the initial TFL and TWP scale developed in the study.

### 3.3 Data collection (two) and reanalysis of measures

#### 3.3.1 Data collection and analysis

The questionnaires were generated electronically with the help of the Questionnaire Star platform after being reviewed and tested on a small sample by experts from the GDUST. The questionnaires were distributed to the target universities using mainstream communication platforms (QQ, WeChat, etc.) with the help of the authors’ classmates, colleagues, and friends. The distribution started from the beginning of July 2023 to the end of 20 August 2023. A total of 403 samples were collected from 11 universities in seven regions of China. All data were entered and coded using SPSS 26.0, and the 11 samples numbered 391, 162, 194, 347, 203, 263, 225, 193, 144, 341, and 254 were removed using data-identification of outlier cases, yielding a usable sample of 392, with an effective rate of recovery of 97.27%. The results showed that the demographic variables covered gender, age, education level, region, education level, Job title, and working period as a teacher. The specific analysis is shown in Table 2. We can see that the proportion of men and women is 37.5% and 62.5%, respectively, and the proportion of women is relatively high. In terms of age, the proportion of the sample aged 31–40 was the largest, 48.7%, the proportion of the sample aged 30 and below was 14.5%, and the proportion of the sample aged 41–50 was 24.2%, which means that the age of the sample was mainly young and middle-aged people around 40 years old. In terms of education, master’s degree accounted for the largest sample size of 68.6%, bachelor’s degree accounted for the second largest sample size of 23.2%, and the total number of samples with master’s degree and above reached 74.5%, so the overall sample of the research is highly educated. In terms of distribution regions, the sample size of South China is the largest, at 62.8%, followed by Northeast China, at 17.1%, while other regions account for a relatively small percentage. In terms of titles, the sample size of lecturers was the largest, accounting for 53.6%, and the sample size of associate professors was the second largest, accounting for 21.9%, with 81.9% of the overall research sample having intermediate and above titles. In terms of the number of years of employment of teachers, the largest number of teachers were 11–20 years, 39%, 21.2% were 6–10 years, and 16.1% were less than 5 years. 20–30 years was similar to the sample size of more than 30 years.

**TABLE 2:** Summary of demographic characteristics.

Demography	Category	Frequency	Percent
Gender	Male	147	37.5
	Female	245	62.5
Age	21∼30	57	14.5
	31∼40	191	48.7
	41∼50	95	24.2
	51∼60	42	10.7
	60+	7	1.8
Education	Below bachelor	9	2.3
	Bachelor	91	23.2
	Master	269	68.6
	Doctor	23	5.9
Region	Northwest	30	7.7
	North	10	2.6
	East	17	4.3
	Southwest	14	3.6
	Central China	8	2
	South	246	62.8
	Northeast	67	17.1
Job title	Teaching assistant	71	18.1
	Lecturer	210	53.6
	Associate professor	86	21.9
	Professor	25	6.4
Working period as a teacher	Less one year	36	9.2
	1–5 years	63	16.1
	6–10 years	83	21.2
	11–20 years	153	39
	20–30 years	31	7.9
	30+	26	6.6

To sum up, the sample of this study has a female bias, which is consistent with the actual situation of most universities. age is the most valued and promising group in the labor market, with more young and middle-aged people, and the respondents generally have a good educational background and a high overall job title level.

#### 3.3.2 Reliability analysis

The internal reliability consistency of the 11 variables of the scale was tested using the reliability test in SPSS 26.0. The Cronbach’s alpha value of the total TFL scale is 0.964, and the alpha coefficients of the six subscales are 0.891 (OV), 0.883 (IC), 0.899 (IS), 0.938 (SL), 0.924 (PR), and 0.918 (MM), greater than 0.7, indicating that the TFL scale’s reliability is high. The Cronbach’s alpha coefficient value for the TWP is 0.954, and the alpha coefficients of the five subscales are 0.908 (IP), 0.913 (LP), 0.833 (HOTC), 0.817 (WD), 0.905 (TSI) all greater than 0.7, indicating that the reliability of the TWP scale is high. And the alpha coefficient of the whole scale is 0.971.

#### 3.3.3 Validity analysis

##### 3.3.3.1 Validity analysis of TFL

Construct validity refers to the degree of consistency between theoretical constructs and measurement scales, that is, the degree of consistency between the content of variable measurement and the definition of the construct (Schwab, 2013). Construct validity includes two aspects: convergent validity and discriminant validity. In terms of testing convergent validity, Hair (2009) suggested that tests should be carried out from three aspects: standardized factor loading, combined reliability and average refined variance. First, the standardization factor loading (STD) should be greater than 0.5. Second, the composition reliability (CR) should be greater than 0.7. Finally, Fornell and Larcker (1981) suggested that the standard value of average variance extracted (AVE) should be greater than 0.5. A confirmatory factor analysis of the TFL was conducted using AMOS 24.0.

Table 3 shows that χ^2^/df = 2.110, which is less than 3; the absolute fit indicators GFI = 0.927, AGFI = 0.901, and RMSEA = 0.053; the relative fit indicators CFI = 0.976, IFI = 0.976, and TLI = 0.971. All of these indices exceeded the standards suggested by Hair et al. (2010), indicating that the TFL scale was acceptable for measurement (Kelloway and Santor, 1999).

**TABLE 3:** Convergent validity test for TFL (*N* = 392).

LV	Item	UNSTD	*t*-value	*P*	STD	1-SMC	CR	AVE	Fit metrics
OV	OV5	1			0.826	0.318	0.895	0.682	X^2^ = 327.108 df = 155 X^2^/df = 2.110 GFI = 0.927 AGFI = 0.901 CFI = 0.976 RMSEA = 0.053 IFI = 0.976 TLI = 0.971
	OV4	1.149	21.026	***	0.881	0.224			
	OV3	1.145	19.214	***	0.828	0.314			
	OV2	1.088	17.152	***	0.764	0.416			
IC	IC5	1			0.86	0.26	0.882	0.714	
	IC3	0.965	20.543	***	0.833	0.306			
	IC2	1.018	20.886	***	0.842	0.291			
IS	IS4	1			0.827	0.316	0.899	0.748	
	IS3	1.093	21.088	***	0.882	0.222			
	IS2	1.104	21.164	***	0.885	0.217			
SL	SL3	1			0.884	0.219	0.94	0.839	
	SL2	1.008	29.006	***	0.938	0.12			
	SL1	1.021	28.13	***	0.925	0.144			
PR	PR3	1			0.878	0.229	0.925	0.805	
	PR2	0.981	25.932	***	0.908	0.176			
	PR1	1.033	25.805	***	0.906	0.179			
MM	MM5	1			0.813	0.339	0.92	0.743	
	MM4	1.182	21.68	***	0.899	0.192			
	MM3	1.216	21.223	***	0.886	0.215			
	MM1	1.204	19.787	***	0.846	0.284			

*** indicates *P* < 0.001.

In the table, all the CR values take more than 0.5, indicating that the latent variable performs well in terms of internal uniformity among the measurement entries. All the factor loadings (STD) also took values over 0.7, the *t*-values took well over 2.0, and the AVE’s took values over 0.5, indicating that the latent variables performed well in terms of convergent validity. The analysis can conclude that the scale of TFL expresses excellent fitting criteria and good convergent reliability with good validity.

##### 3.3.3.2 Constructing a second-order model of TFL

TFL has more constructs and a more complex structure, and it is difficult for the first-order model to fully realize the research purpose. Baek et al. (2018) constructed a second-order model for the more classical MLQ-6S and proved the reliability of the conclusions and the validity of the method. Therefore, this paper attempts to construct a second-order model of TFL. The fitness indicators of the model are shown in Table 4. All the fitting indicators of Model 3 and Model 4 are within the acceptable range, indicating that both models fit well and can be analyzed in the next research. The target coefficient *T* = 327.108/463.268 = 0.706. The results show that the second-order model of TFL is generally effective as an alternative to the first-order model, but the model can be considered for use in general situations.

**TABLE 4:** Fit indices of second-order validation factors for TFL.

Second-order factor model	χ^2^	df	χ^2^/df	GFI	AGFI	CFI	RMSEA
0. NULL model	7,431.496	190	39.113	0.135	0.044	0	0.312
1. One-order one-factor analysis	1,964.764	170	11.557	0.584	0.486	0.752	0.164
2. First-order six-factor model (no correlation between factors)	2,028.363	170	11.932	0.576	0.476	0.749	0.167
3. First-order six-factor model (correlation between the factors)	327.108	155	2.110	0.927	0.901	0.976	0.053
4. Second-order factor model	463.268	164	2.825	0.892	0.862	0.959	0.068

##### 3.3.3.3 Validity analysis of TWP

The results of the validated factor analysis of the five components of TWP using the same methodology as TFL are shown in Table 5.

**TABLE 5:** Convergent validity test of TWP (*N* = 392).

LV	Item	UNSTD	*t*-value	*P*	STD	1-SMC	CR	AVE	Fit metrics
IP	IP1	1			0.843	0.289	0.907	0.71	χ^2^ = 264.249 df = 109 χ^2^/df = 2.424 GFI = 0.927 AGFI = 0.898 CFI = 0.970 RMSEA = 0.060 IFI = 0.970 TLI = 0.963
	IP2	0.932	19.126	***	0.804	0.354			
	IP3	1.01	22.853	***	0.901	0.188			
	IP4	0.972	19.715	***	0.82	0.328			
LP	LP1	1			0.817	0.333	0.914	0.726	
	LP2	1.039	20.19	***	0.857	0.266			
	LP3	1.071	21.199	***	0.885	0.217			
	LP4	0.954	19.939	***	0.849	0.279			
HOTC	HOTC1	1			0.798	0.363	0.833	0.624	
	HOTC3	1.115	16.618	***	0.78	0.392			
	HOTC4	1.103	16.96	***	0.792	0.373			
WD	WD2	1			0.796	0.366	0.822	0.606	
	WD3	0.845	16.704	***	0.79	0.376			
	WD4	0.928	15.614	***	0.748	0.44			
TSI	TSI1	1			0.879	0.227	0.907	0.764	
	TSI2	1.004	24.362	***	0.895	0.199			
	TSI4	0.934	22.171	***	0.848	0.281			

*** indicates *P* < 0.001.

As before, the χ^2^/df = 2.424. The Absolute Fit Indicator, GFI = 0.927 which is within the ideal range of values. AGFI = 0.898, RMSEA = 0.060 which are within a reasonable range of values and close to the ideal value. The relative fit metrics CFI = 0.970, IFI = 0.970, and TLI = 0.963; these metrics are all within the ideal range of values. Overall, this indicates that the data fit the model well enough to proceed to the next step in the calculations. All the values of CR, STD, and AVE in the table exceeded the corresponding criteria, which indicates that the TWP Scale has good validity.

##### 3.3.3.4 Construction of a second-order model of TWP

A second-order model of the TWP was constructed using the same approach as TFL. The values of the fitness indicators for all models are shown in Table 6. Each of the fitted indicators for model 7 and model 8 are within the acceptable range. The objective coefficient *T* = 264.249/302.235 = 0.87. The high value of T indicates that the second-order model of TWP can be analyzed instead of the first-order model.

**TABLE 6:** Fit indicators for second-order validation factors of TWP.

Second-order factor model	χ^2^	df	χ^2^/df	GFI	AGFI	CFI	RMSEA
0. NULL model	5,350.766	136	39.344	0.165	0.061	0	0.313
1. One-order one-factor analysis	1,097.348	119	9.221	0.696	0.609	0.812	0.145
2. First-order five-factor model (no correlation between factors)	1,593.870	119	13.394	0.620	0.512	0.717	0.178
3. First-order five-factor model (correlation between the factors)	264.249	109	2.424	0.927	0.898	0.970	0.060
4. Second-order factor model	302.235	114	2.651	0.917	0.889	0.964	0.065

#### 3.3.4 Common method biases

The data need to be tested for common method biases (CMB) before analysis. To ensure the objectivity and accuracy of the results, this study uses a variety of scientific methods to test the CMB of the collected data. Firstly, using Harman’s single factor test method, all variables in the study were loaded into the exploratory factor analysis, and the maximum factor interpretation variation did not reach 50% of the total variation of the common factor interpretation of all feature roots greater than 1 in the case of rotation, which can be preliminarily judged that there is a CMB in this study, but it is not significant. Considering the low statistical effect of Harman’s single factor test method in detecting CMB, according to Podsakoff et al. (2003) study, the CMB was further tested by controlling the untested single method latent variable method, and the effect of the common method was incorporated into the seven-factor model proposed in this study as a latent variable, and the difference in fitting degree between the two models before and after the inclusion of the common method latent variable was compared, the result is shown in Table 7. It can be seen that the 8-factor model incorporating the latent variable of the common method deviation is better than the 7-factor model before the inclusion, but the changes in RMSEA, CFI, GFI and AGFI are weak, and the degree of improvement does not exceed 0.02, which indicates that the model fit does not improve significantly after the addition of the homologous method deviation factor (Williams et al., 1989). It can be seen that although the influence of CMB cannot be excluded in this study, the CMB is not serious and will not adversely affect the subsequent analysis.

**TABLE 7:** Controlling untested single methods for testing CMB.

Model	χ^2^	Df	χ^2^/df	GFI	AGFI	CFI	RMSEA
7 Factors	1,171.716	603	1.943	0.861	0.838	0.956	0.049
8 Factors	1,029.085	566	1.818	0.878	0.849	0.964	0.046
| Δ|	142.631	37	0.125	0.017	0.011	0.008	0.003

## 4 Findings of the study

### 4.1 TFL in Chinese private universities

Quantitative data analysis shows that teachers generally found that their deans are practicing TFL, but not to a particularly high degree. Because the mean of each characteristic of TFL is above 3.6309 (Table 8), and the overall mean is 3.8697. The means of the variables reflect that deans of secondary colleges in private universities do better in TFL in terms of OV and IC, followed by PR of faculty members, followed in order by MM, IS, and SL.

**TABLE 8:** Descriptive analysis of each latent variable of TFL (*N* = 392).

LV	Minimum	Maximum	Mean	Std. deviation	Skewness		Kurtosis	
					Statistic	Std. error	Statistic	Std. error
OV	1.5	5	3.9628	0.6881	−0.038	0.123	−0.452	0.246
IC	2	5	3.9992	0.70459	−0.219	0.123	−0.524	0.246
IS	2	5	3.8214	0.72926	−0.026	0.123	−0.538	0.246
SL	1	5	3.6309	0.88106	−0.35	0.123	0.098	0.246
PR	2	5	3.9217	0.69073	−0.18	0.123	−0.249	0.246
MM	1	5	3.8561	0.79649	−0.398	0.123	−0.017	0.246
TFL	2	5	3.8697	0.03215	0.101	0.123	−0.567	0.246

Based on the interviews, deans and department chairs appear to prefer IC, organizational goals, and PR (Dean 1; Director 1;). Only the president, vice president, and human resources manager (President Liang, GDUST; Manager Zhang, Human Resources; Vice President Zhao) mentioned IS, SL, and MM.

This evidence suggests that the behavior of transformational leaders is related to the level of leadership. The lower the hierarchy of leadership, the less TFL behavior is exhibited.

In addition, the interview data found that TFL is rapidly growing in private universities. Because leaders are actively exploring and practicing educational philosophy, teaching quality, innovation capability, international development, social responsibility, and win-win cooperation to promote private universities’ sustainable development and improvement. These findings verify the first hypothesis.

### 4.2 The status of TWP in private universities

Table 9 shows that WD and TSI have higher scores than others, followed by LP, while the results for IP and HOTC are relatively lower.

**TABLE 9:** Descriptive analysis of each variable of TWP (*N* = 392).

LV	Minimum	Maximum	Mean	Std. deviation	Skewness		Kurtosis	
					Statistic	Std. E	Statistic	Std. E
IP	1.5	5	3.7823	0.72057	0.117	0.123	−0.408	0.246
LP	2.25	5	4.0215	0.62433	0.101	0.123	−0.695	0.246
HOTC	2	5	3.785	0.689	0.25	0.123	−0.571	0.246
WD	2.67	5	4.1014	0.58537	−0.006	0.123	−0.64	0.246
TSI	2.67	5	4.1042	0.63631	−0.07	0.123	−0.809	0.246
TWP	2.65	5	3.9407	0.02872	0.268	0.123	−0.473	0.246

Std. E = Std. error.

Based on the interview data, it was found that leaders at different levels have different perceptions of TWP. From the responses of leaders at different levels of leadership, it is clear that the principal of the school cares about the development of teachers, while leaders at other levels are influenced by their work tasks and consider the performance of teachers in terms of the development of students as well as the school.

The assessment of these aspects of performance needs to be conducted in conjunction with specific job criteria and considering the actual work of teachers to ensure fairness and accuracy of the assessment. The study found that TWP in private universities in China has relatively high WD and TSI scores, but there is much potential for improvement in IP, LP, and HOTC.

### 4.3 The Relationship Between TFL and TWP

#### 4.3.1 Correlation analysis

In this study, Pearson correlation coefficient analysis was conducted using SPSS 26.0 for 11 dimensions of the two variables, and the results were obtained as shown in Table 10.

**TABLE 10:** Correlation analysis between variables.

	OV	IC	IS	SL	PR	MM	TFL	IP	LP	HOTC	WD	TSI	TWP
OV	1												
IC	0.741**	1											
IS	0.658**	0.725**	1										
SL	0.592**	0.620**	0.620**	1									
PR	0.647**	0.693**	0.643**	0.738**	1								
MM	0.613**	0.620**	0.607**	0.784**	0.739**	1							
TFL	0.834**	0.847**	0.819**	0.861**	0.866**	0.873**	1						
IP	0.549**	0.471**	0.499**	0.458**	0.505**	0.445**	0.570**	1					
LP	0.563**	0.510**	0.505**	0.417**	0.489**	0.419**	0.563**	0.686**	1				
HOTC	0.616**	0.556**	0.570**	0.509**	0.541**	0.478**	0.637**	0.685**	0.706**	1			
WD	0.558**	0.569**	0.550**	0.418**	0.521**	0.437**	0.591**	0.590**	0.712**	0.721**	1		
TSI	0.491**	0.472**	0.482**	0.360**	0.443**	0.339**	0.501**	0.559**	0.707**	0.687**	0.774**	1	
TWP	0.646**	0.592**	0.603**	0.504**	0.582**	0.497**	0.665**	0.847**	0.888**	0.878**	0.850**	0.835**	1

**Correlation is significant at the 0.01 level (2-tailed).

The results of the correlation analysis showed that TWP and TFL (*r* = 0.665, *p* = 0.000 < 0.050), OV (*r* = 0.646, *p* = 0.000 < 0.050), IC (*r* = 0.592, *p* = 0.000 < 0.050), IS (*r* = 0.603, *p* = 0.000 < 0.050), and SL (*r* = 0.504, *p* = 0.000 < 0.050), and PR (*r* = 0.582, *p* = 0.000 < 0.050) are significantly positively and moderately correlated; TWP and MM of transformational leader (*r* = 0.497, *p* = 0.000 < 0.050) are significantly and positively and weakly correlated. From the dimensional analysis of TFL and TWP, the coefficients are distributed between 0.339 and 0.616, indicating a significant moderate or low positive correlation between the dimensions.

#### 4.3.2 Hypothetical test

**FIGURE 3:**
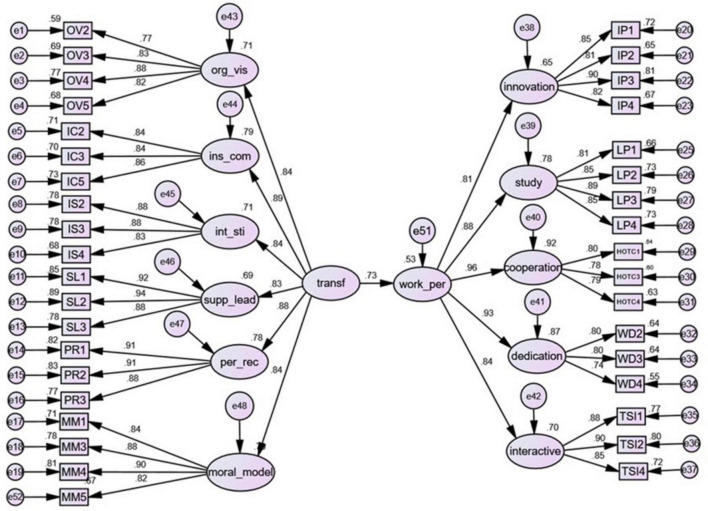
Model 1 second-order TFL and TWP.

After the previous CFA analysis, the structural equation model (SEM) 1 was constructed as shown in Figure 3, which contains the result of the operation. The structure of both TFL and TWP used a second-order model instead of a first-order model to simplify the overall model structure.

There are many calculation methods for SEM. When the sample obeys normal distribution, the most commonly used maximum likelihood method is used for calculation. The calculated results show that the fitted values of the model the χ^2^ and CFI are in the ideal range, and the remaining metric values are within the acceptable range. Data is available in Supplementary Table 1.

Therefore, the result of accepting the output of the model, that is, the path coefficient of TFL and TWP shows that for every standard unit added by TFL, the TWP can increase by 0.73 units, which verifies the second hypothesis.

**FIGURE 4:**
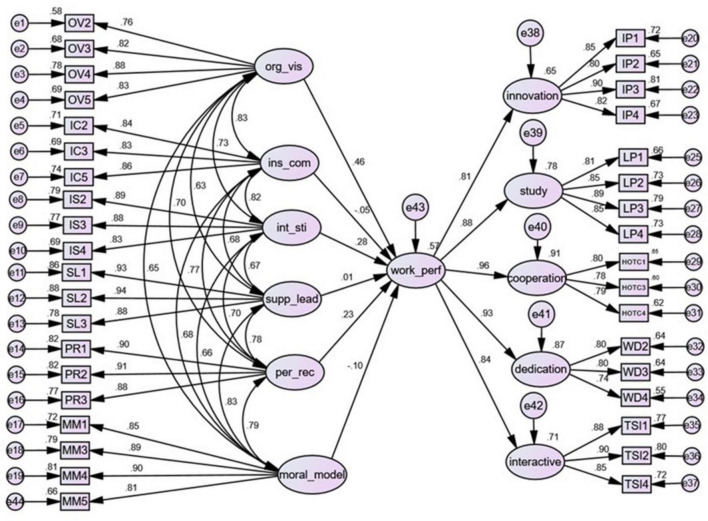
Model 2 TFL and second-order TWP.

To verify which specific aspects of TFL affect TWP, SEM model 2 was constructed as shown in Figure 4.

The results showed that the fit of the model GFI and AGFI are within the acceptable range, and other indicators are within the ideal value range, indicating that the data and the model fit well (Data is available in Supplementary Table 2). The model has a certain explanatory power. Among them, the path test results of Model 2 showed that only the three hypotheses of the effect of OV, IS, and PR on TWP are validated.

The path coefficient results indicated that for every standard deviation increased in the OV, the TWP increased by 0.46 standard deviations; for each additional standard deviation in IS, the TWP increased by 0.28 standard deviations; and for each additional standard deviation increased by PR, the TWP increased by 0.23 standard deviations. The results showed that OV has a medium effect on TWP, and IS, PR has a small effect on it. The effect of IC, SL, MM on TWP is not significant. The results are shown in Supplementary Table 3.

In examining the link between TFL and TWP in private universities, the study supports the positive impact of TFL on TWP. This will complement the literature on how TFL affects the TWP in private universities in China. In addition, this study further reveals the effectiveness of leadership and the management methods used by leaders through conversations with leaders at all levels of private universities.

### 4.4 Recommendations for improving TWP based on AMO theory from TFL perspective

The performance of teachers can have different effects on educational institutions, students, and teachers themselves. In addition, the outcome of teachers’ work output, such as talents and research results, also have an important impact on society. According to quantitative studies, there is space to improve the TWP in private universities in China. Therefore, it is necessary to propose recommendations to improve TWP based on AMO theory from the perspective of TFL to provide value to the aforementioned stakeholders.

**FIGURE 5:**
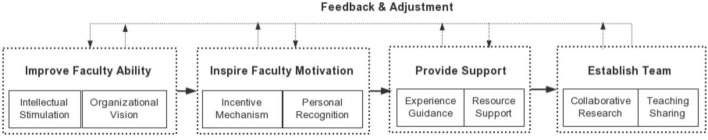
Recommendations framework for improving TWP.

Based on the interview keywords extracted from the respondents’ effectiveness in motivating teachers and leadership experience of treat teachers survey. Then, based on the statistics of these frequent words, this study proposes the following framework to facilitate the improvement of TWP under the TFL perspective in private universities in China, as shown in Figure 5.

Within a TFL perspective, educational leaders can use the framework of AMO theory to promote TWP, which in turn drives development and change in educational organizations.

Specifically, characteristics of TFL include stimulating creativity and innovation, supporting staff self-development and growth, encouraging staff participation in decision-making and problem-solving, and driving organizational development and change. This leadership style is consistent with the framework of AMO theory and can promote TWP by enhancing teachers’ competencies, motivating them, and providing good opportunities.

For example, transformational leaders can enhance teachers’ competencies by providing appropriate education and training to help them improve their teaching skills and subject knowledge. In addition, by providing rewards and recognition mechanisms, teachers can be motivated and increase their enthusiasm and willingness to work. At the same time, improving teaching resources and facilities and providing better opportunities and conditions for teachers are conducive to their ability and motivation, which in turn enhances their performance.

Thus, a link between TFL, AMO theory, and TWP is formed, which can help educational leaders develop more effective management strategies and ways to enhance TWP.

## 5 Conclusion and further research

### 5.1 Conclusion

The role of transformational leadership in universities is becoming an important research topic in the context of changes in digital education and the construction of “double first-class.” This work explores transformational leadership in private universities in China and the impact of transformational leadership on teachers’ work performance, and constructs a framework for improving teacher performance from a transformational leadership perspective.

First, hypothesis 1 was proved, which is the same as the research of Zhang et al. (2009). It suggests that transformational leadership exists in private universities in China, but at different levels and presenting different characteristics. This is very meaningful for the sustainable development of private universities.

Second, the establishment of Hypothesis 2 proved that transformational leadership had a positive impact on teachers’ work performance, which was the same as the research of Andriani et al. (2018), Guan (2020), and Lai et al. (2020). In-depth research found that some characteristics of transformational leadership have a positive impact on teachers’ work performance, such as OV, IS, and PR. (1) Organizational vision has a positive and significant effect on teachers’ work performance, which is the same as the views and findings of Dermol and Širca (2018), Slåtten et al. (2021), and Shao et al. (2022). It showed that transformational leaders use organizational vision to stimulate the potential of teachers to achieve their goals, so that in addition to accomplishing things within their roles, they are willing to do things that are beneficial to school development, thus improving performance such as learning and innovation. (2) Intellectual stimulation has a positive and significant effect on teachers’ work performance, which is the same as some of the findings of Çekmecelioğlu and Özbağ (2016) and Koh et al. (2019). It showed that the intellectual stimulation of transformational leaders encourages teachers to try new ways of doing things and to apply new knowledge, such as teaching methods, teaching information technology, etc., thus improving their performance in learning, innovation, and collaboration with others. (3) Personal recognition has a positive and significant effect on teachers’ work performance, which is the same as the research of Conzelmann (2020). At the same time this conclusion confirms part of the conclusions of Li-Chaoping (2005), that is: individualized care has a prominent impact on employees’ job performance, of which it has the greatest impact on work dedication. It fully proves the effectiveness of personal recognition which was separated from the individualized care dimension as an independent dimension of transformational leadership. (4) While the positive impact of inspirational communication, supportive leadership, and moral modeling by transformational leaders on teachers’ work performance have not been proven. However, correlation analyses show that they are moderately or weakly positive correlation with teachers’ job performance. This suggests that there is some variability in the role of the dimensions of transformational leadership, which can be further investigated subsequently.

Finally, based on the qualitative analysis of the interviews with leaders, this study conducted frequency counts of keywords, mainly related to personal quality, mentoring students, innovation, caring for staff needs, focusing on staff development, providing opportunities, mentoring teachers, rewarding, and forming teams. As a result, this study proposes a framework of recommendations for improving performance, including improving teachers’ competencies, motivating teachers, providing good work opportunities and environments, and building good teacher teams. Specifically:

(1) Improving faculty ability

Transformational leaders make the skills gaps in achieving the vision clear to teachers by establishing the vision, communicating the vision, and providing training, communication, and mentorship through leadership modeling to close the competency gaps (A). In addition, transformational leaders should help teachers continually improve their teaching competencies and professionalism to adapt to changing educational needs. At the same time, teachers are encouraged to explore new teaching methods and techniques that will enhance their competencies and teaching (IS).

(2) Inspire faculty motivation

Transformational leaders should provide teachers with challenging teaching tasks and encourage them to try new teaching methods and techniques, thus motivating teachers to work and be creative. At the same time, transformational leaders should establish transparent incentives, such as pay, promotion, recognition, and incentive trips, to motivate teachers to work and be creative. In addition, personal recognition can play a positive role when teachers take on challenging tasks.

(3) Provide good work opportunities and environment

Transformational leaders should provide teachers with appropriate teaching resources, technical equipment and management support, create a positive learning atmosphere, and improve teachers’ work efficiency and job satisfaction. At the same time, teachers should be encouraged to communicate with their peers and explore new ideas and methods of teaching together, thus improving their professionalism and innovation.

(4) Build good teacher teams

Transformational leaders should build teams of teachers to work together on research and teaching sharing to promote interaction and communication among teachers and improve their professionalism and innovation. At the same time, transformational leaders should encourage teachers to bring out their individual strengths in teaching to improve the quality and effectiveness of teaching.

In short, transformational leaders should take full advantage of the TFL characteristics according to the development stage of the organization, and take effective measures to enhance teachers’ abilities, motivate them, provide a good working environment, and build their teams to ultimately improve their performance.

### 5.2 Further research

While most Western studies show that idealizing influence, inspirational motivation, and individualized care are the most frequently cited dimensions of transformational leadership, this research shows that organizational vision, intellectual stimulation, and personal recognition are the most common behaviors of transformational leadership. This may explain the psychological needs of high-knowledge workers, a desire to grow and be recognized. Future research focusing on these dimensions may help explain transformational leadership structures for high-knowledge workers. At the same time, the sample of this study is only from private universities in China. In the future, we can further compare and analyze whether there is a difference in the impact of transformational leadership on public and private teachers under specific cultural backgrounds.

Regarding how transformational leadership affects teachers’ work performance, this research shows that organizational vision is a common behavior affecting teachers’ work performance most of the time. In other words, when there are no significant gaps in other conditions (e.g., pay), the potential for growth in private institutions is a key factor in faculty members’ willingness to work and strive for the organization, and the potential for growth needs to be tapped by both leaders and teachers. The results of the study found the important role of organizational vision, and also found that Chinese private universities still need to further improve the expression of organizational vision. Future research could explore how private universities in China implement organizational change frameworks central to organizational vision.

In this research, there is no substantiated view that inspirational communication, supportive leadership and moral modeling affect teachers’ work performance. Concerning inspirational communication, theoretically, it should have some impact on the implementation of organizational vision, and future research could explore the impact of inspirational communication on organizational vision and teachers’ work performance. This is a more in-depth study, which can further reveal the influence mechanism of transformational leadership. while supportive leadership, further exploration of whether supportive leadership is truly “transformative” is a left-over question. And in China, does supportive leadership apply? These issues require further research to resolve. As for the morality model, it has a profound historical origin in Chinese education. However, organizational management, it may be influenced by deep-rooted Confucian cultural ideas, making power distance weaken its role in change leadership. Future research could explore whether moral modeling affects teachers’ work performance through other mediating factors, such as: “relationship” or “emotional intelligence.”

## Data availability statement

The original contributions presented in this study are included in this article/Supplementary material, further inquiries can be directed to the corresponding authors.

## Ethics statement

Ethical review and approval was not required for the study on human participants in accordance with the local legislation and institutional requirements. Written informed consent from the patients/participants or patients/participants legal guardian/next of kin was not required to participate in this study in accordance with the national legislation and the institutional requirements.

## Author contributions

XY: Conceptualization, Investigation, Methodology, Writing – original draft, Writing – review & editing. GJ: Resources, Writing – review & editing.

## References

[B1] Al-Husseini S. Elbeltagi I. (2018). Evaluating the effect of transformational leadership on knowledge sharing using structural equation modelling: The case of Iraqi higher education. *Int. J. Leadersh. Educ.* 21 506–517. 10.1080/13603124.2016.1142119

[B2] Al-Husseini S. El Beltagi I. Moizer J. (2021). Transformational leadership and innovation: The mediating role of knowledge sharing amongst higher education faculty. *Int. J. Leadersh. Educ.* 24 670–693. 10.1080/13603124.2019.1588381

[B3] Alimo-Metcalfe B. Alban-Metcalfe R. J. (2001). The development of a new transformational leadership questionnaire. *J. Occup. Organ. Psychol.* 74 1–27. 10.1348/096317901167208

[B4] Andriani S. Kesumawati N. Kristiawan M. (2018). The influence of the transformational leadership and work motivation on teachers performance. *Int. J. Sci. Technol. Res.* 7 19–29.

[B5] Baek H. Byers E. H. Vito G. F. (2018). Transformational leadership and organizational commitment in Korean police station: Test of second-order MLQ-6 S and OCQ. *Int. J. Police Sci. Manag.* 20 155–170. 10.1177/1461355718774582

[B6] Bagozzi R. Yi Y. (1988). On the evaluation of structural equation models. *J. Acad. Mark. Sci.* 143 74–94. 10.1007/BF02723327

[B7] Bass B. M. (1985). *Leadership and performance beyond expectations.* New York, NY: The Free Press.

[B8] Bass B. M. (1996). *New paradigm of leadership: An inquiry into transformational leadership*. US Army Research Institute for the Behavioral and Social Sciences.

[B9] Bass B. M. Riggio R. E. (2006). *Transformational leadership*, 2nd Edn. New York, NY: Psychology Press. 10.4324/9781410617095

[B10] Bass B. M. Waldman D. A. Avolio B. J. Bebb M. (1987). Transformational leadership and the falling dominoes effect. *Group Organ. Stud.* 12 73–87. 10.1177/105960118701200106

[B11] Begum N. Begum S. Rustam A. Rustam S. (2018). Gender perspectives of TFL style and leadership effectiveness: A case study of Pakistan and Turkey. *Dialogue* 13:211–224.

[B12] Bono J. E. Foldes H. J. Vinson G. Muros J. P. (2007). Workplace emotions: The role of supervision and leadership. *J. Appl. Psychol.* 92:1357. 10.1037/0021-9010.92.5.1357 17845090

[B13] Borman W. C. Motowidlo S. J. (1997). Task performance and contextual performance: The meaning for personnel selection research. *Hum. Perform*. 10, 99–109. 10.1207/s15327043hup1002_3

[B14] Buil I. Martínez E. Matute J. (2019). Transformational leadership and employee performance: The role of identification, engagement and proactive personality. *Int. J. Hosp. Manag.* 77 64–75. 10.1016/j.ijhm.2018.06.014

[B15] Burns J. M. (1978). *Leadership.* New York, NY: Harper & Row, 11–12.

[B16] Carlson D. S. Kacmar K. M. Williams L. J. (2000). Construction and initial validation of a multidimensional measure of work–family conflict. *J. Vocation. Behav.* 56 249–276. 10.1006/jvbe.1999.1713

[B17] Çekmecelioğlu H. G. Özbağ G. K. (2016). Leadership and creativity: The impact of transformational leadership on individual creativity. *Proc. Soc. Behav. Sci.* 235 243–249. 10.1016/j.sbspro.2016.11.020

[B18] Churchill G. A. Jr. (1979). A paradigm for developing better measures of marketing constructs. *J. Mark. Res.* 16 64–73. 10.1177/002224377901600110

[B19] Conzelmann J. D. (2020). Leaders recognizing and rewarding organizational citizenship behaviours during formal employee performance evaluations. *eJ. Soc. Behav. Res. Bus.* 11 21–38.

[B20] Dai M. Shi Q. Chen C. (2017). An empirical study on the structure model of teachers’ job performance in colleges and universities. *Stat. Sci.* 12 90–91.

[B21] De Massis A. Frattini F. Lichtenthaler U. (2013). Research on technological innovation in family firms: Present debates and future directions. *Fam. Bus. Rev.* 26 10–31. 10.1177/0894486512466258

[B22] Dermol V. Širca N. T. (2018). Communication, company mission, organizational values, and company performance. *Proc. Soc. Behav. Sci.* 238 542–551. 10.1016/j.sbspro.2018.04.034

[B23] Eisenhardt K. (1989). Building theories from case studies. *Acad. Manag. Rev.* 14 532–550. 10.2307/258557

[B24] Eliyana A. Ma’arif S. (2019). Job satisfaction and organizational commitment effect in the transformational leadership towards employee performance. *Eur. Res. Manag. Bus. Econ.* 25 144–150. 10.1016/j.iedeen.2019.05.001

[B25] Fornell C. Larcker D. F. (1981). Evaluating structural equation models with unobservable variables and measurement error. *J. Mark. Res.* 18 39–50. 10.1177/002224378101800104

[B26] Gilbert S. L. Kelloway E. K. (2018). Leadership, recognition and well-being: A moderated mediational model. *Can. J. Admin. Sci.* 35 523–534. 10.1002/cjas.1477

[B27] Givens R. J. (2008). Transformational leadership: The impact on organizational and personal outcomes. *Emerg. Leadersh. J.* 1 4–24.

[B28] Griffin M. A. Neal A. Parker S. K. (2007). A new model of work role performance: Positive behavior in uncertain and interdependent contexts. *Acad. Manag. J.* 50 327–347. 10.5465/amj.2007.24634438

[B29] Guan Y. (2020). The influence of transformational leadership on the work performance of university administrators. *China Adult Educ.* 06 37–41.

[B30] Gumusluoglu L. Ilsev A. (2009). Transformational leadership, creativity, and organizational innovation. *J. Bus. Res.* 62 461–473. 10.1016/j.jbusres.2007.07.032

[B31] Hair J. F. (2009). *Multivariate data analysis.* Faculty and Research Publications.

[B32] Hair J. F. Black W. C. Babin B. J. Anderson R. E. (2010). *Multivariate data analysis*, 7th Edn. Upper Saddle River, NJ: Prentice Hall.

[B33] Han Y. Liao J. Long L. (2007). Model construction and empirical research of employee job performance structure. *J. Manag. Sci.* 10 62–77.

[B34] House R. J. (1998). Measures and assessments for the charismatic leadership approach: Scales, latent constructs, loadings, Cronbach alphas, and interclass correlations. *Monogr. Organ. Behav. Ind. Relat.* 24 23–30.

[B35] Huang Y. T. Liu H. Huang L. (2021). How transformational and contingent reward leaderships influence university faculty’s organizational commitment: The mediating effect of psychological empowerment. *Stud. High. Educ.* 46 2473–2490. 10.1080/03075079.2020.1723534

[B36] Ilgen D. R. Hollenbeck J. R. Johnson M. Jundt D. (2005). Teams in organizations: From input-process-output models to IMOI models. *Annu. Rev. Psychol.* 56 517–543. 10.1146/annurev.psych.56.091103.070250 15709945

[B37] Jantz R. C. (2017). Vision, innovation, and leadership in research libraries. *Library Inform. Sci. Res.* 39 234–241. 10.1016/j.lisr.2017.07.006

[B38] Jaskyte K. (2004). Transformational leadership, organizational culture, and innovativeness in nonprofit organizations. *Nonprofit Manag. Leadersh.* 15 153–168. 10.1002/nml.59

[B39] Jisun N. (2009). The components and role of a shared vision. *Enterprise Reform Manag.* 2 54–55.

[B40] Judge T. A. Piccolo R. F. (2004). Transformational and transactional leadership: A meta-analytic test of their relative validity. *J. Appl. Psychol.* 89 755. 10.1037/0021-9010.89.5.755 15506858

[B41] Kalsoom Z. Khan M. A. Zubair D. S. S. (2018). Impact of transactional leadership and transformational leadership on employee performance: A case of FMCG industry of Pakistan. *Ind. Eng. Let.* 8 23–30.

[B42] Kammerhoff J. Lauenstein O. Schütz A. (2019). Leading toward harmony–Different types of conflict mediate how followers’ perceptions of transformational leadership are related to job satisfaction and performance. *Eur. Manag. J.* 37 210–221. 10.1016/j.emj.2018.06.003

[B43] Kassarjian H. H. (1977). Content analysis in consumer research. *J. Consumer Res.* 4 8–18. 10.1086/208674

[B44] Kelloway E. K. Santor D. A. (1999). Using LISREL for structural equation modelling: A researcher’s guide. *Can. Psychol.* 40:381. 10.1037/h0092500

[B45] Koh D. Lee K. Joshi K. (2019). Transformational leadership and creativity: A meta-analytic review and identification of an integrated model. *J. Organ. Behav.* 40 625–650. 10.1002/job.2355

[B46] Lai F. Y. Tang H. C. Lu S. C. Lee Y. C. Lin C. C. (2020). Transformational leadership and job performance: The mediating role of work engagement. *Sage Open* 10:2158244019899085. 10.1177/2158244019899085

[B47] Lawrason S. V. Shaw R. B. Turnnidge J. Côté J. (2023). Characteristics of transformational leadership development programs: A scoping review. *Eval. Program Plann.* 101:102354. 10.1016/j.evalprogplan.2023.102354 37611362

[B48] Lee A. Legood A. Hughes D. Tian A. W. Newman A. Knight C. (2020). Leadership, creativity and innovation: A meta-analytic review. *Eur. J. Work Organ. Psychol.* 29 1–35. 10.1080/1359432X.2019.1661837

[B49] Li N. Jung G. Y. Kim H. H. (2020). A study on the effect of transformation leadership on the job performance of employees by AMO model. *J. Korea Converg. Soc.* 11 41–50.

[B50] Li-Chaoping S. K. (2005). The structure and measurement of transformational leadership in China. *Acta Psychol. Sin.* 37 803–811.

[B51] Liu P. (2018). Transformational leadership research in China (2005–2015). *Chin. Educ. Soc.* 51 372–409. 10.1080/10611932.2018.1510690

[B52] London M. Mone E. M. (1999). “Continuous learning,” in *The changing nature of performance: Implications for staffing, motivation, and development*, ed. Pulakos (San Francisco, CA: Jossey-Bass Publishers), 119–153.

[B53] London M. Sessa V. I. (2006). Group feedback for continuous learning. *Hum. Resour. Dev. Rev*. 5, 303–329. 10.1177/1534484306290226

[B54] Manuel E. G. (2021). Inspirational Leadership, Innovation and Entrepreneurship. *Effect. Exec.* 24 38–42.

[B55] McCoach D. B. Gable R. K. Madura J. P. (2013). *Instrument development in the affective domain*, Vol. 10. New York, NY: Springer, 971–978.

[B56] Metwally A. H. El-Bishbishy N. Nawar Y. S. (2014). The impact of transformational leadership style on employee satisfaction. *Bus. Manag. Rev.* 5 32–42.

[B57] Mitchell R. Boyle B. (2019). Inspirational leadership, positive mood, and team innovation: A moderated mediation investigation into the pivotal role of professional salience. *Hum. Resour. Manag.* 58 269–283. 10.1002/hrm.21951

[B58] O’Connell D. Hickerson K. Pillutla A. (2011). Organizational visioning: An integrative review. *Group Organ. Manag.* 36 103–125. 10.1177/1059601110390999

[B59] Piccolo R. F. Colquitt J. A. (2006). Transformational leadership and job behaviors: The mediating role of core job characteristics. *Acad. Manag. J.* 49 327–340. 10.5465/amj.2006.20786079

[B60] Podsakoff P. M. MacKenzie S. B. Lee J. Y. Podsakoff N. P. (2003). Common method biases in behavioral research: A critical review of the literature and recommended remedies. *J. Appl. Psychol.* 88:879. 10.1037/0021-9010.88.5.879 14516251

[B61] Podsakoff P. M. MacKenzie S. B. Moorman R. H. Fetter R. (1990). Transformational leader behaviors and their effects on followers’ trust in leader, satisfaction, and organizational citizenship behaviors. *Leadersh. Q.* 1 107–142. 10.1016/1048-9843(90)90009-7

[B62] Rafferty A. E. Griffin M. A. (2004). Dimensions of transformational leadership: Conceptual and empirical extensions. *Leadersh. Q.* 15 329–354. 10.1016/j.leaqua.2004.02.009

[B63] Rukundo A. Akurut C. R. (2021). Association between inspirational leadership traits and job commitment among secondary school teachers in a remote district of Uganda. *Can. J. Educ. Soc. Stud.* 1 46–62. 10.53103/cjess.v1i1.4

[B64] Salas-Vallina A. Simone C. Fernández-Guerrero R. (2020). The human side of leadership: Inspirational leadership effects on follower characteristics and happiness at work (HAW). *J. Bus. Res.* 107 162–171. 10.1016/j.jbusres.2018.10.044

[B65] Schwab D. P. (2013). *Research methods for organizational studies.* London: Psychology Press. 10.4324/9781410611284

[B66] Seitz S. R. Owens B. P. (2021). Transformable? A multi-dimensional exploration of transformational leadership and follower implicit person theories. *Eur. J. Work Organ. Psychol.* 30 95–109. 10.1080/1359432X.2020.1830761

[B67] Shao H. Fu H. Ge Y. Jia W. Li Z. Wang J. (2022). Moderating effects of transformational leadership, affective commitment, job performance, and job insecurity. *Front. Psychol.* 13:847147. 10.3389/fpsyg.2022.847147 35615161 PMC9125335

[B68] Singh M. (2019). Teaching competence of prospective teachers in relation to their digital literacy inspirational leadership and creative intelligence. Available online at: http://localhost:12345/GET?f=URLfAltAct.htm&URL=http%3A%2F%2Fshodhganga.inflibnet.ac.in%2F&CategoryGroup=Entertainment&NoReplace=1

[B69] Slåtten T. Lien G. Evenstad S. B. N. Onshus T. (2021). Supportive study climate and academic performance among university students: The role of psychological capital, positive emotions and study engagement. *Int. J. Q. Serv. Sci.* 13 585–600. 10.1108/IJQSS-03-2020-0045

[B70] Song H. (2010). *The influence of transformational leadership behavior on job performance and its mechanism.* Berlin: Harbin Institute of Technology.

[B71] Strike V. M. Michel A. Kammerlander N. (2018). Unpacking the black box of family business advising: Insights from psychology. *Fam. Bus. Rev.* 31 80–124. 10.1177/0894486517735169

[B72] Tamsah H. Nasaruddin N. Tahir S. Z. B. (2021). “The Role of Inspirational Leadership in Improving the Work Quality of Employees of the Tomakaka Education Group in Mamuju,” in *Proceedings of the 11th Annual International Conference on Industrial Engineering and Operations Management*. Universitas Iqra Buru, 7176–7187.

[B73] Thrash T. M. Elliot A. J. Maruskin L. A. Cassidy S. E. (2010). Inspiration and the promotion of well-being: Tests of causality and mediation. *J. Pers. Soc. Psychol.* 98:488. 10.1037/a0017906 20175626

[B74] Top C. Abdullah B. M. S. Faraj A. H. M. (2020). Transformational leadership impact on employees performance. *Eur. J. Manag. Soc. Sci.* 1 49–59. 10.23918/ejmss.v1i1p49

[B75] Wenyi D. (2016). “A study of the impact of transformational leadership on the job performance of young physical education teachers in higher education,” in *Proceedings of 2016 6th International conference on education and sports education (ESE 2016 V52)* (Kuala Lumpur: Singapore Management and Sports Science Institute), 19–22.

[B76] Williams L. J. Cote J. A. Buckley M. R. (1989). Lack of method variance in self-reported affect and perceptions at work: Reality or artifact? *J. Appl. Psychol.* 74:462. 10.1037//0021-9010.74.3.462

[B77] Yin R. K. (2009). *Case study research–; design and methods*, 4th Edn. Thousand Oaks, CA: Sage.

[B78] Zhang Y. Sun H. Hu Q. Wang L. (2009). A survey of transformational leadership behavior in colleges and universities: Based on a typical case analysis of a general college in Shandong Province. *Manag. Observ.* 16 148–151.

