# Supplementary material for: A framework for transformational leadership to enhance teacher’s work performance
Source: Front Psychol. 2024 Jul 23;15:1331597. doi: 10.3389/fpsyg.2024.1331597 (PMC11300363; doi:10.3389/fpsyg.2024.1331597)
Supplement: Supplementary file 1 [file Data_Sheet_1.docx]

Supplementary Material

# Supplementary Figures


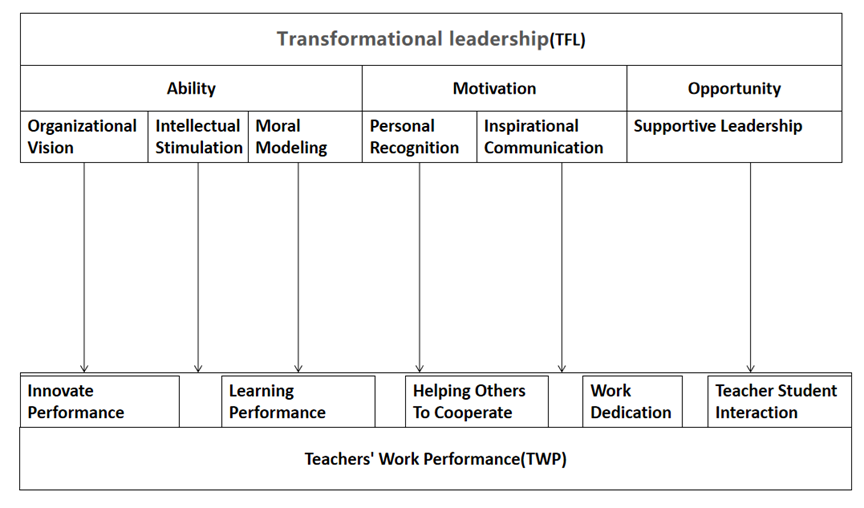


Supplementary Figure 1. AMO Links TFL with TWP

Supplementary Table 1. Parameter Calculation and Model Fitting Index Value of Model 1

| **studied variables** | | | **Estimate** | **S.E.** | **C.R.** | **P** | **Path Coefficients** |
| --- | --- | --- | --- | --- | --- | --- | --- |
| work_per | <--- | transf | 0.753 | 0.071 | 10.678 | *** | 0.729 |
| org_vis | <--- | transf | 1 |  |  |  | 0.843 |
| ins_com | <--- | transf | 1.102 | 0.08 | 13.693 | *** | 0.891 |
| int_sti | <--- | transf | 1.101 | 0.081 | 13.612 | *** | 0.842 |
| supp_lead | <--- | transf | 1.304 | 0.094 | 13.94 | *** | 0.829 |
| per_rec | <--- | transf | 1.098 | 0.076 | 14.413 | *** | 0.881 |
| moral_model | <--- | transf | 1.215 | 0.092 | 13.166 | *** | 0.835 |
| innovation | <--- | work_per | 1 |  |  |  | 0.806 |
| study | <--- | work_per | 0.913 | 0.066 | 13.9 | *** | 0.883 |
| cooperation | <--- | work_per | 0.993 | 0.069 | 14.433 | *** | 0.957 |
| dedication | <--- | work_per | 1.009 | 0.072 | 14.056 | *** | 0.934 |
| interactive | <--- | work_per | 0.917 | 0.065 | 14.082 | *** | 0.839 |
| **Fitted index value** | | | χ^2^=1344.854；df=617；χ^2^/df=2.180；GFI=0.840；AGFI=0.818；  CFI=0.944；RMSEA=0.055 | | | | |

Supplementary Table 2. Parameter Calculation and Model Fitting Index Value of Model 2

| **studied variables** | | | **Path Coefficients** | **Estimate** | **S.E.** | **C.R.** | **P** | **NH** |
| --- | --- | --- | --- | --- | --- | --- | --- | --- |
| work_perf | <--- | org_vis | 0.46 | 0.402 | 0.083 | 4.858 | *** | S |
| work_perf | <--- | ins_com | -0.05 | -0.043 | 0.1 | -0.426 | 0.67 | NS |
| work_perf | <--- | int_sti | 0.28 | 0.217 | 0.067 | 3.22 | 0.001** | S |
| work_perf | <--- | supp_lead | 0.01 | 0.004 | 0.056 | 0.066 | 0.948 | NS |
| work_perf | <--- | per_rec | 0.23 | 0.188 | 0.075 | 2.504 | 0.012* | S |
| work_perf | <--- | moral_model | -0.10 | -0.071 | 0.064 | -1.112 | 0.266 | NS |
| **Fitted index value** | | | χ^2^=1171.716; df=603; χ^2^/df=1.943; GFI=0.861; AGFI=0.838; CFI=0.956; RMSEA=0.049 | | | | | |

Noted:* means significant at 0.05 alpha level, ** means significant at 0.01 level, ***P<0.001 level significant; NH=Null Hypothesis ;S=support; NS= Not support

Supplementary Table 3. Summary of Hypothesis Testing Results of TFL on TWP

| **Hypothetical Content** | **Test Result** |
| --- | --- |
| H1：TFL has been displayed in private universities in China. | Support |
| H2: TFL has a positive impact on TWP in private universities and colleges in China. | Support |
| H2a: Organization vision of transformational leaders have an impact on work performance of teachers in private universities and colleges in China. | Support |
| H2b：Inspirational communication of transformational leaders has a positive impact on work performance of teachers in private universities and colleges in China. | No Support |
| H2c：Intellectual simulation of transformational leaders has a positive impact on work performance of teachers in private universities and colleges in China. | Support |
| H2d：Supportive leadership of transformational leaders has a positive impact on work performance of teachers in private universities and colleges in China. | No Support |
| H2e：Personal recognition of transformational leaders has a positive impact on work performance of teachers in private universities and colleges in China. | Support |
| H2f：Moral modeling of transformational leaders has a positive impact on work performance of teachers in private universities and colleges in China. | No Support |
